# Periacetabular osteotomy with and without concomitant arthroscopy: a systematic review of evidence on post-operative activity levels and return to sport

**DOI:** 10.1093/jhps/hnad043

**Published:** 2023-11-29

**Authors:** Phillip Wyatt, Sarah Cole, James Satalich, Brady S Ernst, John Cyrus, Alexander Vap, Robert O’Connell

**Affiliations:** Virginia Commonwealth University School of Medicine, 1000 E Marshall St, Richmond, VA 23298, USA; Virginia Commonwealth University School of Medicine, 1000 E Marshall St, Richmond, VA 23298, USA; Department of Orthopedic Surgery, Virginia Commonwealth University Medical Center, 1200 E Broad St, 9th Floor, Box 980153, Richmond, VA 23298, USA; Department of Orthopedic Surgery, Virginia Commonwealth University Medical Center, 1200 E Broad St, 9th Floor, Box 980153, Richmond, VA 23298, USA; Virginia Commonwealth University School of Medicine, 1000 E Marshall St, Richmond, VA 23298, USA; Department of Orthopedic Surgery, Virginia Commonwealth University Medical Center, 1200 E Broad St, 9th Floor, Box 980153, Richmond, VA 23298, USA; Department of Orthopedic Surgery, Virginia Commonwealth University Medical Center, 1200 E Broad St, 9th Floor, Box 980153, Richmond, VA 23298, USA

## Abstract

The purpose of this systematic review is to (i) compare post-operative activity levels after periacetabular osteotomy (PAO) versus PAO + HA (concomitant PAO and hip arthroscopy) using patient-reported outcomes that specifically assess activity and sports participation [Hip Disability and Osteoarthritis Outcome Score—Sport and Recreation subscale (HOOS-SR), University of California Los Angeles (UCLA) activity score, Hip Outcome Score—Sport-Specific Subscale (HOS-SSS)] and (ii) compare post-operative return to sport (RTS) data between PAO and PAO + HA groups. A systematic review of literature was conducted on 1 June 2023, utilizing PubMed, Cochrane and Embase (OVID). Articles were screened for inclusion using specific inclusion and exclusion criteria. Twenty-six out of 1610 articles met all inclusion criteria, without meeting any exclusion criteria. In the 12 studies containing only subjects who underwent PAO alone, 11 demonstrated an average score improvement in UCLA, HOOS-SR or HOS-SSS post-operatively (*P* < 0.05). In the three studies containing subjects who underwent PAO with concomitant HA, significant improvements were seen in the HOS-SS and UCLA scores (*P* < 0.05). In the five studies that directly compared UCLA, HOS-SSS and HOOS-SSS scores between PAO groups and PAO + HA groups, all demonstrated statistically significant improvement post-operatively (*P* < 0.05). The rate of RTS ranged from 63% to 90.8% among PAO studies and was found to be 81% in the single PAO + HA study that assessed RTS. When performed in patients with intra-articular pathology, concomitant PAO + HA may provide similar sport-related outcomes to PAO alone in patients without intra-articular pathology.

## INTRODUCTION

Periacetabular osteotomy (PAO) is a well-studied method to treat patients with symptomatic hip dysplasia. This surgical technique aims to conserve joint cartilage within the hip via acetabular reorientation and femoral stabilization [1–3]. Prior studies have shown that the primary demographic group for hip dysplasia consists of young females in the age range of 14–18 years and that the condition is common within the female athletic community [4, 5]. As hip pain and groin pain have shown to be prevalent obstacles in athletes [6, 7], PAO is an effective tool in improving sports participation and activity levels in hip dysplasia patients [8–17].

Although PAO effectively corrects for malalignment within the hip joint, an increasing number of studies have demonstrated a high prevalence of intra-articular pathology within the dysplastic hip that may require arthroscopic intervention [18]. While not recommended as an isolated treatment in moderate and severe cases of hip dysplasia [19], arthroscopic techniques have been used in select patients with mild dysplasia, but they remain controversial in their efficacy [20]. Patients with untreated but identified concomitant intra-articular pathology often have worse surgical outcomes following a PAO and may undergo arthroscopic intervention in the future [21–23]. Furthermore, there is evidence that a previously failed HA may worsen outcomes in patients who later undergo PAO [24]. These discoveries have created the need for further research on the effectiveness of a combined PAO and HA (PAO + HA) in managing patients with hip dysplasia—particularly in young and active groups. Favorable activity-related outcomes have been found in these patients [25–30].

Several prior systematic reviews have assessed outcomes of PAO and/or PAO + HA. One study found that the majority of patient-reported outcomes (PROs) improved post-operatively when included and that conversion rates to total hip arthroplasty (THA) were lower in arthroscopy alone compared with PAO and PAO with arthroscopy [31]. However, since their publication, at least eight more studies have investigated outcomes of concomitant PAO and HA, analyzing the impact of this surgery on sports participation and the activity level, and have overall seemed to find no significant differences in PROs between PAO and PAO + HA [26–28, 30, 32–35]. Additionally, this study failed to include PROs such as University of California Los Angeles (UCLA) activity score or hip disability and osteoarthritis outcome score—sports and recreation (HOOS-SR) or data regarding return to sport (RTS) in athletic populations. Six more systematic reviews have looked at outcomes of PAO [36–41], with one assessing RTS outcomes without the inclusion of PROs [37]. This particular study found that PAO is an effective procedure in athletes to improve sports participation post-operatively. The other studies have overall found good clinical outcomes in a variety of categories related to hip survivorship after an isolated PAO [37–40]. However, none of these studies compared results to groups receiving PAO + HA [36–41]. Finally, another systematic review assessed outcomes and survivorship after PAO + HA, finding favorable outcomes overall, without comparing their data to an isolated PAO [42].

The purpose of this systematic review is to (i) compare post-operative activity levels after PAO versus PAO + HA using PROs that specifically assess activity and sports participation [(HOOS-SR, UCLA activity score and Hip Outcome Score—Sport-Specific Subscale (HOS-SSS)] and (ii) compare post-operative RTS data between PAO and PAO + HA groups. Our research question is as follows: in subjects with dysplastic hips, do concomitant PAO and HA, when compared to an isolated PAO, result in improved post-operative sports and activity outcomes? Our hypothesis is that sports and activity outcomes will be similar in patients who underwent PAO alone or PAO with concomitant arthroscopy.

## METHODS

### Search strategy

This study is a systematic review of studies published prior to 1 June 2023 that have investigated RTS and/or post-operative activity levels in subjects who have undergone PAO or PAO + HA. This study followed the Preferred Reporting Items for Systematic Reviews and Meta-Analyses (PRISMA) guidelines for reporting systematic reviews [43]. One author searched PubMed/Medline, Cochrane and Embase (OVID) databases on 1 June 2023, using the following terms:(((hip OR acetabular OR‘Acetabulum’[MeSH]) AND (dysplasia OR dysplastic))OR ‘Hip Dislocation’[MeSH])AND (periacetabular AND (osteotomy OR‘Osteotomy’[MeSH])).

### Summary of the screening process

Duplicates and papers written in languages other than English were excluded. Two authors independently screened all articles returned from the initial search using pre-determined inclusion and exclusion criteria. Each study was first screened for relevance by title and abstract. Full text for studies deemed ‘relevant’ was then reviewed for inclusion/exclusion in this systematic review. Conflicts were resolved by a neutral third author.

Inclusion criteria are as follows: (i) human studies that either report outcomes in patients who underwent PAO alone or report outcomes in patients who underwent concomitant PAO and arthroscopy, (ii) studies that report outcomes at least 1 year post-operatively, (iii) surgeries must be indicated for hip dysplasia and (iv) studies that report RTS data or sport/activity-related outcomes according to any of the following three PRO measures: HOS-SSS, Hip Disability and Osteoarthritis Outcome Score—Sport and Recreational Activity (HOOS-SR) and University of California Los Angeles (UCLA) activity scale. The HOS-SSS is a validated sub-scale used to assess RTS/activity after hip surgery [44, 45], including within the context of hip dysplasia [46, 47]. The HOOS-SR has been validated and used in the post-THA population [48, 49] as well as in patients who have undergone PAO[50]. The UCLA activity score serves as a general estimate of patients’ post-operative activity levels that have been validated for monitoring physical activity levels in those with hip and/or knee osteoarthritis [51]; it has also been used in studies on hip dysplasia [52, 53].

Exclusion criteria are as follows: case reports, review articles, conference abstract presentations, non-English studies, studies that do not report outcomes at least 1 year post-operatively, studies that do not explicitly state whether a PAO in isolation or a combined PAO/arthroscopy was performed, studies that contain both PAO and PAO + HA subjects but do not provide separate analyses for both populations, non-human studies, any indication for surgery other than hip dysplasia, studies investigating only modified PAO techniques, studies that did not report on RTS or any of the following three PRO measures: HOS-SSS, HOOS-SR and UCLA activity score.

### Quality assessment

Two authors assessed each included study for quality and risk of bias. For case-control studies and cohort studies, the Newcastle–Ottawa Scale (NOS) was used [54]. Case series studies were assessed using the National Institute of Health (NIH) Quality Assessment Tool for case series studies and prospective designs with before–after studies without a control group [55].

### Outcomes of interest and data extraction

Outcomes of interest included RTS data, HOS-SSS scores, HOOS-SR scores and UCLA activity scores. Data were extracted from each of the included studies by two authors independently using a standardized data extraction table created by one of the authors. Relevant information related to each study’s methods and subject demographics was collected in addition to outcomes of interest.

## RESULTS

### Search results

**Fig. 1.**
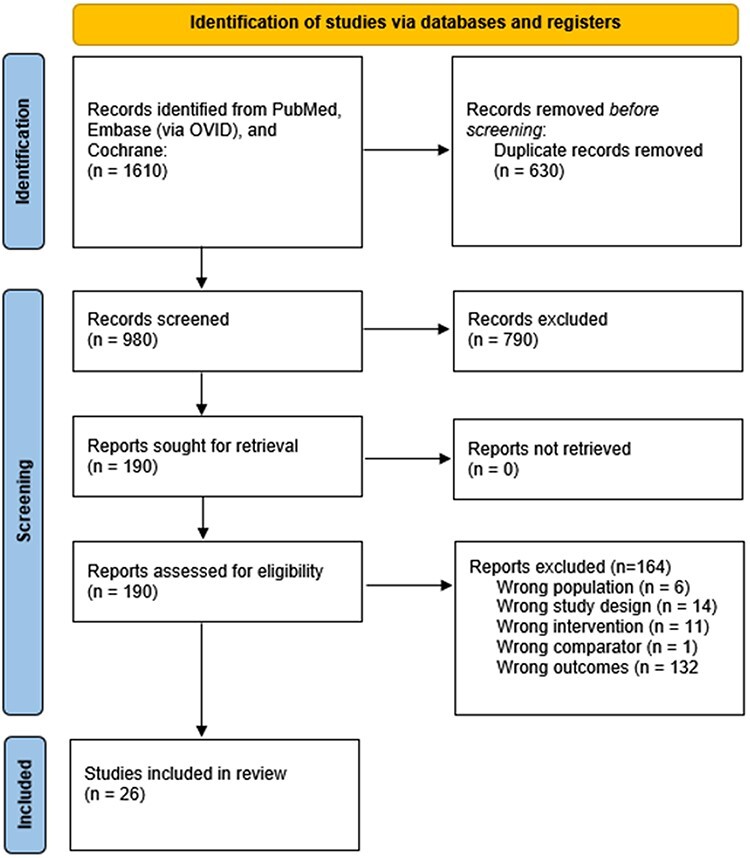
PRISMA flow diagram. There were 32 studies included in this systematic review.

The initial search returned 1610 articles. After 630 duplicates were removed, there were 980 studies left to be screened by title and abstract. Of these, 790 were excluded based on inclusion/exclusion criteria. There were 190 full-text articles sought for retrieval, all of which were successfully retrieved. Of these, a total of 165 articles were excluded based on inclusion/exclusion criteria, leaving a total of 26 studies that met inclusion criteria without any exclusion criteria [8–14, 16, 17, 26–28, 30, 32–35, 50, 53, 56–62]. The inclusion/exclusion process is depicted in Fig. 1.

### Study design and quality assessment

Thirteen of the 26 included studies were case series design [8–14, 16, 26, 27, 32, 35, 56], three were case-control design [10, 28, 58], six were retrospective cohort design [30, 33, 34, 53, 59, 60] and four were prospective cohort design [50, 57, 61, 62]. Eleven out of 13 case series studies were determined to be of ‘good’ quality with at least seven out of nine NIH criteria being met. One case series met only four out of nine NIH criteria due to poorly described statistical methods and lack of detailed description of pre-operative injury and functional status of their subjects [9]. All three case-control studies met eight out of eight NOS criteria and were considered to be of ‘good’ quality [10, 28, 58]. Eight of the 10 cohort studies met at least eight out of eight NOS criteria and were considered to be of ‘good’ quality. Two prospective cohort studies lacked a control group and therefore were evaluated with the NIH quality assessment tool for before–after studies without the control group. They met nine out of nine criteria and were also determined to be of ‘good’ quality [50, 57].

### Characteristics of studies

In the 26 included studies, 82.3% of the subjects were female and the average age at the time of surgery was 27.7 years. The average length of follow-up among the included studies was 3.52 years (range, 1–18 years). Across 15 studies, there were 1523 subjects (1544 hips) who received a PAO alone [8–14, 16, 17, 50, 53, 56, 57, 59, 62]. Across 10 studies, there were 355 subjects (360 hips) who received concomitant PAO + HA [26–28, 30, 32–35, 59, 60], six of which directly compared outcomes between PAO and PAO + HA groups [28, 30, 32, 34, 58, 61]. Another one compared outcomes in staged versus concomitant PAO + HA procedures [33]. The remaining three PAO + HA subject groups were found in three case series studies [26, 27, 35]. Comparative groups in other studies included the following: PAO versus THA [53], PAO outcomes in mild versus severe hip dysplasia [62], PAO in sporting subjects versus non-sporting subjects [10] and PAO outcomes in subjects with versus without a history of failed HA [60].

### PRO measures

In the 12 studies containing only subjects who underwent PAO alone, 11 demonstrated an average score improvement in UCLA, HOOS-SR or HOS-SSS post-operatively. The remaining study demonstrated successful return to baseline in UCLA score [8]. Details regarding the included PAO-alone studies can be found in Table I. In the three studies containing subjects who underwent PAO with concomitant HA, significant improvements were seen in the HOS-SSS [27, 35] and UCLA [26] scores. Details regarding studies containing only PAO + HA subjects can be found in Table II. In the five studies that directly compared UCLA, HOS-SSS and HOOS-SSS scores between PAO groups and PAO + HA groups, all demonstrated statistically significant improvement post-operatively [28, 32, 34, 58, 61]. It is worth mentioning that one of the most frequently used PRO measures, the International Hip Outcome Tool (iHOT), was not included in the primary analysis of the present study [63]. Although six of the included studies reported iHOT scores [11, 14, 26, 60–62], the authors chose not to include these data in the primary analysis because the iHOT scores did not report sport/recreation-specific subscores, which is the primary focus of this study. Therefore, it was not included in the primary analysis of the present study. These details regarding these and other included comparative studies can be found in Table III.

**Table I.** Outcomes of interest in studies where subjects received PAO alone

*PAO-alone study*	*Design (n*, *average length of follow-up)*	*UCLA activity score (average)*	*HOOS-SR (average)*	*HOS-SSS (average)*	*RTS and other activity-related data*
Heyworth et al. (2016)^[1]^	Case series (*n* = 41; 3 years)	Pre-operative: 8.2 ± 2 Post-operative: 8.2 ± 2 Δ = 0 (*P* = 0.29)	DNR	DNR	80% of participants RTS at a median time of 9 months (95% CI, 7–11 months) Of the 37 returning athletes, 17 (73%) returned to the same level, although this was significantly less common in competitive athletes (11 of 19; 58%) compared to recreational athletes (16 of 18; 89%) Post-operative pain ratings found to be the only predictive factor associated with time to RTS (*P* = 0.01)
Bergayk and Garbuz (2002)^[2]^	Case series (*n* = 25; 2 years)	DNR	DNR	DNR	Average Tegner and Lysholm scores improved from 1.9 to 4.4 (*P*-value not reported) from pre-operative to post-operative. These scores correlate with an increase from walking and light labor to recreational sports and moderately heavy labor
Swarup et al. (2020)^[3]^	Case series (*n* = 33; 1 year)	DNR	DNR	Pre-operative: 31 (SD 20) Post-operative: 81 (SD 18) Δ = 50 (SD 29; *P* < 0.01) 91% of subjects achieved MCID (+6 points)	DNR
Hara et al. (2017)^[4]^	Case-control (*n* = 161; 8.33 years)	Overall pre-operative: 4.7 ± 2.1 Overall post-operative: 5.5 ± 2.0 Δ = 0.8 (*P* < 0.001) Sporting subjects pre-operative: 5.0 ± 2.3 Sporting subjects post-operative: 6.4 ± 1.9 Δ = 1.4 (*P* < 0.05) Non-sporting subjects pre-operative: 4.3 ± 1.8 Non-sporting subjects post-operative: 4.5 ± 1.5 Significantly higher post-operative score in the sporting group (*P* < 0.03)	DNR	DNR	DNR
Jakobsen et al. (2018)^[5]^	Prospective cohort without the control group (*n* = 146; 2.3 years)	DNR	Pre-operative: 44 (SD 24.1) Post-operative 6 months: 69 (SD 23.3) Post-operative 2 years: 71 (SD 23.4) Δ = 27 (*P* < 0.000)	DNR	DNR
Beaule et al. (2015)^[6]^	Case series (*n* = 67; 5 years)	Pre-operative: 5.3 (SD 2.2) Post-operative: 6.6 (SD 2.6) Δ = 1.3 (95% CI 0.5–2.1; *P* = 0.002)	DNR	DNR	DNR
Boje et al. (2019)^[7]^	Case series (*n* = 321; 2 years)	DNR	Pre-operative: 42.76 (SD 22.92) Post-operative: 69.49 (SD 25.48) Δ = 26.73 (95% CI 23.63–29.83; *P* = 0.000)	DNR	DNR
Leopold et al. (2023)^[8]^	Case series (*n* = 111; 5.25 years)	Pre-operative: 5.08 ± 2.44 Post-operative: 6.95 ± 1.74 Δ = 1.87 (*P* < 0.001)	DNR	DNR	Overall participation in sports increased from 78.8% pre-operative to 90.8% post-operative (*P* = 0.008) Significant increase in participation in low-impact sports from pre-operative to post-operative (31.7% versus 52%; *P* = 0.001). Non-significant decrease in high-impact sports pre-operative to post-operative (42.3% versus 36.6%; *P* = 0.361) At final follow-up, 58.1% reported that the surgery improved their sports ability; 18.8% reported decreased sports ability; 23.1% reported no change in sports ability
Novais et al. (2013)^[9]^	Case series (*n* = 51; 2 years)	Significant increase from pre-operative to post-operative at both 1- and 2-year follow-ups Δ 1 year = 0.9 ± 2.3 (*P* < 0.001) Δ 2 years = 1.0 ± 2.4 (*P* < 0.001)	DNR	DNR	DNR
Novais et al. (2018)^[10]^	Case series (*n* = 33; 2.7 years)	DNR	DNR	DNR	63% returned to dance at an average of 8.8 months
Wasko et al. (2019)^[11]^	Prospective cohort without the control group (*n* = 294; 1 year)	DNR	Pre-operative: 40.9 ± 25.3 Post-operative: 75.4 ± 23.3 Δ = 34.5 (*P* < 0.05) 73% of subjects achieved MCID for HOOS-SR (+12.6 points)	DNR	DNR
Wells et al. (2017)^[12]^	Case series (*n* = 99; 18 years)	Post-operative scores significantly different between asymptomatic and symptomatic hips (*P* = 0.001): Asymptomatic hips: 7 ± 2 Symptomatic hips: 6 ± 2	DNR	DNR	DNR

DNR, did not report; Δ, change in score; HOOS-SR, Hip disability and Osteoarthritis Outcome Scale—Sport and Recreation subscale; MCID, minimal clinically important difference.

**Table II.** Outcomes of interest in studies containing only subjects who received concomitant PAO and HA to address intra-articular pathology

*PAO + HA study*	*Design (n*, *average length of follow-up)*	*UCLA activity score (average)*	*HOOS-SR (average)*	*HOS-SSS (average)*	*RTS and other activity-related data*
Maldonado et al. (2019)^[1]^	Case series (*n* = 16; 5 years)	DNR	DNR	Pre-operative: 37.6 ± 23.9 Post-operative: 68.1 ±23.0 Δ = 30.5 (*P* = 0.001) 50% reached a score of ‘PASS’ (75 points), and 78.6% achieved MCID (+6 points)	DNR
Edelstein et al. (2021)^[2]^	Case series (*n* = 67; 6.5 years)	Pre-operative: 6.5 ± 2.7 Post-operative: 7.5 ± 2.2 Δ = 1.0 (*P* = 0.01)	DNR	DNR	DNR
Jimenez et al. (2022)^[3]^	Case series (*n* = 29; 2.83 years)	DNR	DNR	Pre-operative: 43.3 ± 20.1 Post-operative: 80.2 ± 12.5 Δ = 36.5 ± 22.7 (*P* < 0.001) 79.3% achieved MCID (+10 points)	81% successfully RTS. The rate was higher in recreational athletes compared to competitive athletes (85.6% versus 77.8%)

Δ, change in score; DNR, did not report; HOOS-SR, Hip disability and Osteoarthritis Outcome Scale—Sport and Recreation subscale; MCID, minimal clinically important difference; PASS, patient acceptable symptom state.

**Table III.** Outcomes of interest in the included comparative studies

*Comparative Study*	*Design (n*, *average length of follow-up)*	*Groups compared*	*UCLA activity score (average)*	*HOOS-SR (average)*	*HOS-SSS (average)*	*RTS and other activity-related data*
Ricciardi et al. (2016)^[1]^	Prospective cohort (*n* = 73; 1.9 years)	PAO (*n* = 52) PAO + HA (*n* = 21)	DNR	DNR	PAO Pre-operative: 56 ± 19 6-month post-operative: 68 ± 26 6-month Δ_PAO_ = 12 ± 23 12-month post-operative: 80 ± 23 12-month Δ_PAO_ = 27 ± 22 PAO ± HA Pre-operative: 41 ± 20 6-month post-operative: 54 ± 29 6-month Δ_PAO_ = 12 ± 36 12-month post-operative: 80 ± 23 12 month Δ_PAO+HA_ = 37 ± 28 No significant differences between groups in Δ at 6 months (*P* = 0.95) or 12 months (*P* = 0.19)	DNR
Thanacharoenpanich et al. (2018)^[2]^	Retrospective cohort (*n* = 106; median follow-up: 29 months)	PAO (*n* = 47) PAO + HA (*n* = 17) PAO + arthrotomy (*n* = 42)	DNR	PAO Δ_PAO_ = 25.9 (95% CI 16.33–35.49) PAO ± HA Δ_PAO+HA_ = 35.3 (95% CI 18.35–52.24) PAO ± arthrotomy Δ_PAO+arthrotomy_ = 33.6 (95% CI 22.73–44.53) No significant difference among groups (*P* = 0.36)	DNR	DNR
Panos et al. (2021)^[3]^	Case-control (*n* = 48; 2.5 years)	PAO (*n* = 8) PAO + HA (*n* = 17) PAO + arthrotomy (*n* = 23)	PAO Pre-operative: 7.1 (SD 2.3) Post-operative: 8.0 (SD 2.0) Δ_PAO_ = 1.0 (SD 2.5; *P* = 0.41) PAO ± HA Pre-operative: 6.4 (SD 2.9) Post-operative: 7.0 (SD 2.2) Δ_PAO+HA_ = 0.2 (SD 2.4; *P* = 0.55) PAO ± arthrotomy Pre-operative: 7.3 (SD 2.3) Post-operative: 7.1 (SD 2.4) Δ_PAO+arthrotomy_ = −0.3 (SD 2.8; *P* = 0.65) No significant difference between groups at pre-operative, post-operative or for change in score (*P* = 0.56, *P* = 0.58 and *P* = 0.53, respectively)	PAO Pre-operative: 50.9 (SD 25.4) Post-operative: 80.5 (SD 32.6) Δ_PAO_ = 28.6 (SD 30.8; *P* = 0.063) PAO ± HA Pre-operative: 41.3 (SD 21.5) Post-operative: 70.1 (SD 28.8) Δ_PAO+HA_ = 34.6 (SD 30.6; *P* = 0.004) PAO ± arthrotomy Pre-operative: 44.6 (SD 22.0) Post-operative: 75.3 (SD 30.1) Δ_PAO+arthrotomy_ = 30.3 (SD 32.4; *P* = 0.002) No significant difference between groups at pre-operative, post-operative or for change in score (*P* = 0.64, *P* = 0.74 and *P* = 0.90, respectively)	DNR	DNR
Petrie et al. (2020)^[4]^	Case series with sub-analysis of PAO versus PAO + HA groups (*n* = 359; 3.74 years)	PAO (*n* = 294) PAO + HA (*n* = 65)	Univariable logistic regression revealed no major difference in UCLA activity score changes between PAO and PAO + HA subjects (*P* = 0.6496)	DNR	DNR	DNR
Nepple et al. (2023)^[5]^	Case-control (*n* = 186; 3.3 years)	PAO (*n* = 21) PAO + other (*n* = 165) PAO + HA (*n* = 45) PAO + HA + osteoplasty (*n* = 85) PAO + osteoplasty (*n* = 35)	PAO ± osteotomy without arthroscopy Pre-operative: 7.1 ± 2.6 Post-operative: 7.4 ± 2.0 Δ_PAO_ = 0.3 ± 3.2 PAO ± osteotomy with arthroscopy Pre-operative: 6.2 ± 2.6 Post-operative: 7.5 ± 1.9 Δ_PAO+other_ = 1.3 ± 2.6 Significantly greater baseline score (*P* = 0.043) and score increase (*P* = 0.045) in the group with arthroscopy, but similar scores at final follow-up (0.911)	PAO ± osteotomy without arthroscopy Pre-operative: 46 ± 23 Post-operative: 78 ± 20 Δ_PAO_ = 31 ± 26 PAO ± osteotomy with arthroscopy Pre-operative: 39 ± 25 Post-operative: 78 ± 20 Δ_PAO+other_ = 39 ± 25 No significant differences in scores at any time point (*P* > 0.05)	DNR	DNR
Wyles et al. (2018)^[6]^	Retrospective cohort (*n* = 71; 1 year)	PAO +HA (*n* = 39) PAO + arthrotomy (*n* = 32)	PAO ± HA Δ_PAO+HA_ = 0.9 (SD 2.7) PAO ± arthrotomy Δ_PAO+arthrotomy_ = 1.0 (SD 2.4) Similar change in UCLA activity score between groups (*P* = 0.788)	PAO ± HA Δ_PAO+HA_ = 29.8 (SD 30.2) PAO ± arthrotomy Δ_PAO+arthrotomy_ = 35.2 (SD 30.5) Similar change in HOOS-SR between groups (*P* = 0.056)	DNR	DNR
Orner et al. (2023)^[7]^	Retrospective cohort (*n* = 62; 1.68 years)	Staged PAO + HA (*n* = 22) Combined PAO + HA (*n* = 39)	DNR	DNR	Staged PAO ± HA Pre-operative: 55.9 ± 22.6 3-month post-operative: 48.3 ± 33.6 6-month post-operative: 70.6 ± 26.1 12-mont post-operative: 82.0 ± 19.9 Final post-operative: 79.2 ± 23.1 Δ_final_ = 23.3 Combined PAO ± HA Pre-operative: 47 ± 21.5 3-month post-operative: 36.5 ± 28.3 6-month post-operative: 62.5 ± 29.0 12-month post-operative: 72.4 ± 29.1 Final post-operative: 76.0 ± 24.3 Δ_final_ = 29 No statistically significant difference in scores at pre-operative (*P* = 0.469), 3-month post-operative (*P* = 0.343), 6-month post-operative (*P* = 0.691), 12-month post-operative (*P* = 0.678) No statistically significant difference in % of subjects who achieved MCID (78.6% in the staged group, 75.9% in the combined group; *P* = 0.637) or PASS (69.6% in the staged group, 56.4% in the combined group; *P* = 0.639)	DNR
Ricciardi et al. (2017)^[8]^	Retrospective cohort (*n* = 93; 2 years)	PAO with prior failed arthroscopy (*n* = 22) PAO without prior failed arthroscopy (*n* = 71)	DNR	DNR	Significantly lower score in the group with prior failed arthroscopy at the 1-year follow-up (62 ± 25 versus 85 ± 18; *P* < 0.001)	DNR
Shiomoto et al. (2023)^[9]^	Retrospective cohort (*n* = 60; 12 years)	PAO (*n* = 30) THA (*n* = 30) Both indicated for hip dysplasia	PAO Pre-operative: 4.0 Post-operative: 6.1 Δ_PAO_ = 2.1 THA Pre-operative: 4.0 Post-operative: 5.2 Δ_THA_ = 1.2 No significant difference between the two groups in post-operative scores (*P* = ^[2,3,5]^0.09)	DNR	DNR	DNR
Wirries et al. (2022)^[10]^	Retrospective cohort (*n* = 136; 4.4 years)	PAO (*n* = 34) TPO (*n* = 102)	PAO Pre-operative: 5.0 ± 2.1 Post-operative: 8.1 ± 1.3 Δ_PAO_ = 3.1 (*P* = 0.001) TPO Pre-operative: 4.8 ± 2.1 Post-operative: 7.7 ± 1.4 Δ_PAO_ = 2.9 (*P* < 0.05) No significant difference in post-operative scores between the two groups (*P* > 0.05)	DNR	DNR	VAS fitness level TPO: 6.7 ± 2.6 (pre-operative) ➙ 4.5 ± 1.9 post-operative (Δ = −2.2) PAO: 6.4 ± 2.9 (pre-operative) ➙ 5.6 ± 2.3 (post-operative) No significant differences between groups (*P* > 0.05) Hours of sports/week TPO: 2.7 ± 3.1 (pre-operative) ➙ 4.1 ± 3.2 (post-operative) PAO: 3.3 ± 4.0 (pre-operative) ➙ 4.3 ± 3.7 (post-operative) No significant differences between groups (*P* > 0.05)

Δ, change in score; DNR, did not report; HOOS-SR, Hip disability and Osteoarthritis Outcome Scale—Sport and Recreation subscale; MCID, minimal clinically important difference; PASS, patient acceptable symptom state; TPO, triple pelvic osteotomy.

### RTS

For the purposes of reporting, any RTS data reported by the included studies were included in the analysis of this study. Three PAO-alone studies reported RTS data [8, 14, 56]. In all three, RTS was defined as resumption of organized sport (i.e., performance or competition) at any capacity. The average rate of RTS among these three studies was 77.93% (range, 63–90.8%) with an average time to RTS of 8.9 months. The lowest rate (63%) was found in a study of female dancers [56]. One study found a significant increase in low-impact sport participation post-operatively (31.7–52%; *P* = 0.001) while also demonstrating a non-significant decrease in high-impact sport participation post-operatively (42.3–36.6%; *P* = 0.361) [14].

One PAO + HA study reported an 81% rate of RTS at a final follow-up of 2.83 years [27]. Interestingly, they reported a higher rate of RTS in recreational athletes (85.6%) compared to competitive athletes (77.8%). Another study comparing triple PAO to PAO found no significant differences in visual analog scale (VAS) (0–10 scale with 10 being the worst outcome) fitness level and hours of sport participation per week post-operatively between the two groups (*P* > 0.05) [59].

## DISCUSSION

The limited number of available studies included in this review appears to affirm the hypothesis that no significant difference in sports participation and activity levels between subjects who received PAO and PAO + HA exists. Across all variables assessed in this review, patients in both the PAO and PAO + HA groups demonstrated greater activity and sports participation levels in their post-operative evaluations. However, there was no identifiable trend in differences in the outcomes of interest between patients who underwent one of the two procedures. One comparative study found a statistically significant increase in UCLA score in the PAO + HA group compared to PAO alone [58]. Although the heterogeneity of the studies makes direct comparative analysis difficult, the overall results support the use of both procedures to improve sports and activity outcomes in patients with hip dysplasia without a significant variation in their success to do so.

These review findings are agreeable with Lodhia et al., in their conclusion that PRO scores do not vary significantly between PAO and PAO + HA groups in that both cohorts demonstrate improved outcomes post-operatively [31]. Although they demonstrated similar PRO scores between groups in their study, they did not report on specific sport-related outcome measures such as the HOS-SSS, HOOS-SR or UCLA activity score. Similar to our study, they reported a much larger proportion of subjects who received PAO alone (*n* = 703 hips) to subjects who received PAO + HA (*n* = 17 hips). Over the 7 years since publication of the study by Lodhia et al., more studies have investigated PAO + HA groups, providing the current systematic review with a much larger, albeit still unequal, proportion of PAO + HA to PAO (*n* = 355 hips versus *n* = 1544 hips, respectively). In a recent systematic review on outcomes after concomitant PAO + HA, Lee et al. similarly demonstrated excellent post-operative PRO scores [42]. However, they also did not report on sport-specific subscales. Our study builds on the work of these two prior systematic reviews, providing evidence that PAO + HA, when performed on dysplastic hips with intra-articular pathology, leads to similar post-operative sport-related PRO scores compared to subjects who appropriately received PAO without intra-articular intervention [31, 42].

Our findings are in agreement with findings of Curley et al. that PAO can provide good outcomes regarding RTS[36]. Unlike the present review, the authors did not report on subjects with PAO + HA or on sport-specific PROs. However, the rate of RTS found in the present systematic review (63–90.8%) is similar to the rates reported by Curley et al. [36]. This is likely due to the fact that both reviews share multiple PAO studies in common [8, 10, 27, 56, 59]. In addition to these shared studies, our systematic review also reveals data from the study by Leopold et al. that demonstrated a significant decrease in high-impact sports participation from pre-operative to post-operative (42.3% to 36.6%; *P* = 0.361) and an increase in low-impact sports participation from pre-operative to post-operative (31.7% to 52%; *P* = 0.001) [14]. This brings up an interesting point that sports participation after PAO or PAO + HA will largely depend on the target sport/activity. Heyworth et al. similarly found that recreational athletes return to prior level of competition at a higher rate (89%) compared to competitive athletes (college, high school and professional levels) (58%) after PAO [8]. Therefore, when considering RTS expectations in athletic populations, it is important to consider the goal sport and level at which it is hoped to be played post-operatively.

These findings coincide with current supportive evidence for the use of PAO + HA in the case of hip dysplasia with concomitant intra-articular pathology. Prior studies have shown that patients with intra-articular pathology at the time of surgical intervention tend to have longer hip survival when these concomitant issues are addressed in their initial corrective procedure and are less likely to require further surgical treatment later on [21–23]. One study found that advanced intra-articular lesions not addressed during an osteotomy caused a high rate of progression to osteoarthritis in the dysplastic hip, worsening longevity of the procedure [23]. Moreover, several studies have reported low but not insignificant rates of conversion to other surgical procedures, including THA and arthroscopy, in hips that underwent PAO [21, 22, 31, 38]. Without evidence to suggest that PAO + HA will worsen athletic outcomes in cases of active patients with dysplastic hips, the aforementioned results help affirm the use of this procedure in cases with identified intra-articular pathology.

This study is not without limitations. One limitation is the failure of some studies to report any procedures performed concurrently with PAO. This creates the possibility that some cases analyzed as an isolated PAO may actually be properly categorized as PAO + HA (or some other combination of procedures), which could bias comparison between the two procedures. However, this was not standard across all the included studies as many did report concomitant surgeries in both PAO and PAO + HA groups [28, 32, 58, 61]. Heterogeneity in the degree of RTS (i.e., return to the pre-operative level versus return to any level) also limits our conclusions regarding RTS outcomes. Additionally, patients undergoing isolated PAO may have presented with asymptomatic intra-articular pathologies, while those undergoing PAO + HA had symptomatic intra-articular changes. Additionally, several of the included studies did not meet criteria to be considered of ‘good’ quality according to the risk of bias assessment tools used by the authors. Lastly, the studies included in the present systematic review are inherently heterogeneous with varying study protocols, reporting methods and outcome measures. For this reason, the authors determined that a meta-analysis was not feasible. Despite these limitations, this systematic review successfully demonstrated comparable sport- and activity-related outcomes in subjects who undergo PAO and those who undergo PAO + HA.

## CONCLUSION

Based on the heterogeneous studies reported in this systematic review, PAO with and without concomitant HA appears to provide similar sport- and activity-related outcomes. However, the lack of uniform identification of pre-operative intra-articular across studies makes it difficult to conclude that outcomes are truly similar between groups. More high-quality, controlled studies are needed to assess hip survivability in order to help surgeons identify patients who would benefit most from concomitant surgery.

## Data Availability

The data underlying this article will be shared on reasonable request to the corresponding author.

## References

[1] Leunig M, Siebenrock KA, Ganz R. Rationale of periacetabular osteotomy and background work. *Instr Course Lect* 2001; 50: 229–38. 11372318

[2] Ganz R, Klaue K, Vinh TS et al. A new periacetabular osteotomy for the treatment of hip dysplasias. Technique and preliminary results. *Clin Orthop Relat Res* 1988; 232: 26–36. 3383491

[3] Abraham CL, Knight SJ, Peters CL et al. Patient-specific chondrolabral contact mechanics in patients with acetabular dysplasia following treatment with peri-acetabular osteotomy. *Osteoarthr Cartil* 2017; 25: 676–84. 10.1016/j.joca.2016.11.016 PMC6565367 27923602

[4] LaPrade MD, Melugin HP, Hale RF et al. Incidence of hip dysplasia diagnosis in young patients with hip pain: a geographic population cohort analysis. *Orthop J Sports Med* 2021; 9: 2325967121989087. 10.1177/2325967121989087 PMC7940741 33748308

[5] Kapron AL, Peters CL, Aoki SK et al. The prevalence of radiographic findings of structural hip deformities in female collegiate athletes. *Am J Sports Med* 2015; 43: 1324–30. 25828079 10.1177/0363546515576908

[6] Thorborg K, Hölmich P. Advancing hip and groin injury management: from eminence to evidence. *Br J Sports Med* 2013; 47: 602–5. 23407439 10.1136/bjsports-2012-092090

[7] Orchard J, Seward H. Epidemiology of injuries in the Australian Football League, seasons 1997–2000. *Br J Sports Med* 2002; 36: 39–44. 11867491 10.1136/bjsm.36.1.39 PMC1724448

[8] Heyworth BE, Novais EN, Murray K et al. Return to play after periacetabular osteotomy for treatment of acetabular dysplasia in adolescent and young adult athletes. *Am J Sports Med* 2016; 44: 1573–81. 26969123 10.1177/0363546516632743

[9] van Bergayk AB, Garbuz DS. Quality of life and sports-specific outcomes after Bernese periacetabular osteotomy. *J Bone Joint Surg Br* 2002; 84-B: 339–43. 10.1302/0301-620x.84b3.12421 12002489

[10] Hara D, Hamai S, Fukushi J et al. Does participation in sports affect osteoarthritic progression after periacetabular osteotomy?. *Am J Sports Med* 2017; 45: 2468–75. 28586624 10.1177/0363546517707942

[11] Swarup I, Zaltz I, Robustelli S et al. Outcomes of periacetabular osteotomy for borderline hip dysplasia in adolescent patients. *J Hip Preserv Surg* 2020; 7: 249–55. 33163209 10.1093/jhps/hnaa012 PMC7605771

[12] Beaulé PE, Dowding C, Parker G et al. What factors predict improvements in outcomes scores and reoperations after the bernese periacetabular osteotomy?. *Clin Orthop Relat Res* 2015; 473: 615. 10.1007/s11999-014-3980-4 PMC4294893 25287520

[13] Boje J, Caspersen CK, Jakobsen SS et al. Are changes in pain associated with changes in quality of life and hip function 2 years after periacetabular osteotomy? A follow-up study of 321 patients. *J Hip Preserv Surg* 2019; 6: 69–76. 31069098 10.1093/jhps/hnz009 PMC6501443

[14] Leopold VJ, Szarek A, Hipfl C et al. Changes in sports activity after periacetabular osteotomy: a qualitative and quantitative analysis. *Am J Sports Med* 2023; 51: 481–6. 36607176 10.1177/03635465221142320 PMC9909031

[15] Novais EN, Duncan S, Nepple J et al. Do radiographic parameters of dysplasia improve to normal ranges after bernese periacetabular osteotomy?. *Clin Orthop Relat Res* 2017; 475: 1120–7. 27646418 10.1007/s11999-016-5077-8 PMC5339125

[16] Novais EN, Heyworth B, Murray K et al. Physical activity level improves after periacetabular osteotomy for the treatment of symptomatic hip dysplasia. *Clin Orthop Relat Res* 2013; 471: 981. 10.1007/s11999-012-2578-y PMC3563783 23212768

[17] Wells J, Millis M, Kim YJ et al. Survivorship of the bernese periacetabular osteotomy: what factors are associated with long-term failure?. *Clin Orthop Relat Res* 2017; 475: 396–405. 27172819 10.1007/s11999-016-4887-z PMC5213921

[18] Domb BG, Lareau JM, Baydoun H et al. Is intraarticular pathology common in patients with hip dysplasia undergoing periacetabular osteotomy?. *Clin Orthop Relat Res* 2014; 472: 674–80. 24096455 10.1007/s11999-013-3140-2 PMC3890175

[19] Adler KL, Giordano BD. The utility of hip arthroscopy in the setting of acetabular dysplasia: a systematic review. *Arthrosc - J Arthrosc Relat Surg* 2019; 35: 237–48. 10.1016/j.arthro.2018.07.048 30611355

[20] Kirsch JM, Khan M, Bedi A. Does hip arthroscopy have a role in the treatment of developmental hip dysplasia?. *J Arthroplasty* 2017; 32: S28–S31. 28336246 10.1016/j.arth.2017.02.022

[21] Laboudie P, Dymond T, Kreviazuk C et al. Hip arthroscopy after periacetabular osteotomy for acetabular dysplasia – incidence and clinical outcome. *BMC Musculoskelet Disord* 2022; 23: 1–8. 35820874 10.1186/s12891-022-05625-x PMC9275150

[22] Cvetanovich GL, Heyworth BE, Murray K et al. Hip arthroscopy in patients with recurrent pain following Bernese periacetabular osteotomy for acetabular dysplasia: operative findings and clinical outcomes. *J Hip Preserv Surg* 2015; 2: 295–302. 27011852 10.1093/jhps/hnv037 PMC4765306

[23] Fujii M, Nakashima Y, Yamamoto T et al. Effect of intra-articular lesions on the outcome of periacetabular osteotomy in patients with symptomatic hip dysplasia. *J Bone Joint Surg Br* 2011; 93-B: 1449–56. 10.1302/0301-620X.93B11.27314 22058293

[24] Novais EN, Coobs BR, Nepple JJ et al. ANCHOR Study Group. Previous failed hip arthroscopy negatively impacts early patient-reported outcomes of the periacetabular osteotomy: an ANCHOR matched cohort study. *J Hip Preserv Surg* 2018; 5: 370–7. 30647927 10.1093/jhps/hny038 PMC6328744

[25] Cho YJ, Kim KI, Kwak SJ et al. Long-term results of periacetabular rotational osteotomy concomitantly with arthroscopy in adult acetabular dysplasia. *J Arthroplasty* 2020; 35: 2807–12. 32563590 10.1016/j.arth.2020.05.045

[26] Edelstein AI, Nepple JJ, Abu-Amer W et al. What mid-term patient-reported outcome measure scores, reoperations, and complications are associated with concurrent hip arthroscopy and periacetabular osteotomy to treat dysplasia with associated intraarticular abnormalities? *Clin Orthop Relat Res* 2021; 479: 1068–77. 33300755 10.1097/CORR.0000000000001599 PMC8051986

[27] Jimenez AE, Lee MS, Owens JS et al. Athletes undergoing concomitant hip arthroscopy and periacetabular osteotomy demonstrate greater than 80% return-to-sport rate at 2-year minimum follow-up. *Arthroscopy* 2022; 38: 2649–58. 35257741 10.1016/j.arthro.2022.02.017

[28] Panos JA, Gutierrez CN, Wyles CC et al. Addressing intraarticular pathology at the time of anteverting periacetabular osteotomy for acetabular retroversion is associated with better short-term patient-reported outcomes. *J Hip Preserv Surg* 2021; 8: 90–104. 34676101 10.1093/jhps/hnab040 PMC8527802

[29] Sabbag CM, Nepple JJ, Pascual-Garrido C et al. The addition of hip arthroscopy to periacetabular osteotomy does not increase complication rates: a prospective case series. *Am J Sports Med* 2019; 47: 543–51. 30730756 10.1177/0363546518820528

[30] Wyles CC, Hevesi M, Bartels DW et al. Arthroscopy and arthrotomy to address intra-articular pathology during PAO for hip dysplasia demonstrates similar short-term outcomes. *J Hip Preserv Surg* 2018; 5: 282–95. 30393556 10.1093/jhps/hny022 PMC6206691

[31] Lodhia P, Chandrasekaran S, Gui C et al. Open and arthroscopic treatment of adult hip dysplasia: a systematic review. *Arthroscopy* 2016; 32: 374–83. 26507162 10.1016/j.arthro.2015.07.022

[32] Petrie JR, Novais EN, An TW et al. What is the impact of periacetabular osteotomy surgery on patient function and activity levels?. *J Arthroplasty* 2020; 35: S113–8. 32241651 10.1016/j.arth.2020.03.002

[33] Orner CA, Haws BE, Reuter J et al. Patient-reported outcomes are similar in the first two years after staged versus combined hip arthroscopy and periacetabular osteotomy for hip dysplasia. *Arthroscopy* 2023; 39: 1857–65. 36868528 10.1016/j.arthro.2023.02.017

[34] Thanacharoenpanich S, Boyle MJ, Murphy RF et al. Periacetabular osteotomy for developmental hip dysplasia with labral tears: is arthrotomy or arthroscopy required? *J Hip Preserv Surg* 2018; 5: 23–33. 29423247 10.1093/jhps/hnx048 PMC5798119

[35] Maldonado DR, LaReau JM, Perets I et al. Outcomes of hip arthroscopy with concomitant periacetabular osteotomy, minimum 5-year follow-up. *Arthroscopy* 2019; 35: 826–34. 30733041 10.1016/j.arthro.2018.10.143

[36] Curley AJ, Padmanabhan S, Chishti Z et al. Periacetabular osteotomy in athletes with symptomatic hip dysplasia allows for participation in low-, moderate-, and high-impact sports, with greater than 70% return to sport for competitive athletes: a systematic review. *Arthroscopy* 2023; 39: 868–80. 36528217 10.1016/j.arthro.2022.12.004

[37] Curley AJ, Engler ID, Ruh ER et al. Periacetabular osteotomy after failed hip arthroscopy demonstrates improved outcomes in a heterogenous patient population: a systematic review. *Knee Surg Sports Traumatol Arthrosc* 2023; 31: 2090–102. 35974192 10.1007/s00167-022-07108-x

[38] Tan JHI, Tan SHS, Rajoo MS et al. Hip survivorship following the Bernese periacetabular osteotomy for the treatment of acetabular dysplasia: a systematic review and meta-analysis. *Orthop Traumatol: Surg Res* 2022; 108: 103283. 10.1016/j.otsr.2022.103283 35470119

[39] Sohatee MA, Ali M, Khanduja V et al. Does hip preservation surgery prevent arthroplasty? Quantifying the rate of conversion to arthroplasty following hip preservation surgery. *J Hip Preserv Surg* 2020; 7: 168–82. 33163202 10.1093/jhps/hnaa022 PMC7605779

[40] Ahmad SS, Giebel GM, Perka C et al. Survival of the dysplastic hip after periacetabular osteotomy: a meta-analysis. *HIP International* 2023; 33: 306–12. 34569355 10.1177/11207000211048425 PMC9978864

[41] Akhtar M, Razick DI, Wen J et al. Patient-reported outcomes and factors impacting success of the periacetabular osteotomy. *Cureus* 2023; 15 e37320:10.7759/cureus.37320 PMC10167773 37181987

[42] Lee MS, Fong S, Owens JS et al. Outcomes after concomitant hip arthroscopy and periacetabular osteotomy: a systematic review. *Orthop J Sports Med* 2023; 11: 23259671231160560. 10.1177/23259671231160559 PMC10134132 37123992

[43] Page MJ, Moher D, Bossuyt PM et al. PRISMA 2020 explanation and elaboration: updated guidance and exemplars for reporting systematic reviews. *Bmj* 2021; 372: n160. 10.1136/bmj.n160 PMC8005925 33781993

[44] Migliorini F, Baroncini A, Eschweiler J et al. Return to sport after arthroscopic surgery for femoroacetabular impingement. *Surgeon* 2023; 21: 21–30. 34953722 10.1016/j.surge.2021.11.006

[45] Martin RL, Philippon MJ. Evidence of validity for the hip outcome score in hip arthroscopy. *Arthroscopy* 2007; 23: 822–6. 17681202 10.1016/j.arthro.2007.02.004

[46] Della Rocca F, Di Francia V, Schiavi P et al. Hip arthroscopy and T-shaped capsular plication for the treatment of borderline dysplasia: a minimum 2-year follow-up. *Eur J Orthop Surg Traumatol* 2022; 32: 449–58. 34009474 10.1007/s00590-021-02997-z

[47] Domb BG, Stake CE, Lindner D et al. Arthroscopic capsular plication and labral preservation in borderline hip dysplasia: two-year clinical outcomes of a surgical approach to a challenging problem. *Am J Sports Med* 2013; 41: 2591–8. 23956133 10.1177/0363546513499154

[48] Lyman S, Lee YY, McLawhorn AS et al. What are the minimal and substantial improvements in the hoos and koos and jr versions after total joint replacement? *Clin Orthop Relat Res* 2018; 476: 2432–41. 30179951 10.1097/CORR.0000000000000456 PMC6259893

[49] Nilsdotter AK, Lohmander LS, Klässbo M et al. Hip disability and osteoarthritis outcome score (HOOS)—validity and responsiveness in total hip replacement. *BMC Musculoskelet Disord* 2003; 4: 10. 10.1186/1471-2474-4-10 PMC161815 12777182

[50] Wasko MK, Yanik EL, Pascual-Garrido C et al. Psychometric properties of patient-reported outcome measures for periacetabular osteotomy. *J Bone Joint Surg Am* 2019; 101: e21. 10.2106/JBJS.18.00185 30893237

[51] Terwee CB, Bouwmeester W, van Elsland SL et al. Instruments to assess physical activity in patients with osteoarthritis of the hip or knee: a systematic review of measurement properties. *Osteoarthritis Cartilage* 2011; 19: 620–33. 21251989 10.1016/j.joca.2011.01.002

[52] Matheney T, Zaltz I, Kim YJ et al. Activity level and severity of dysplasia predict age at bernese periacetabular osteotomy for symptomatic hip dysplasia. *J Bone Joint Surg Am* 2016; 98: 665–71. 27098325 10.2106/JBJS.15.00735

[53] Shiomoto K, Hamai S, Hara D et al. Objective activity levels and patient-reported outcomes after total hip arthroplasty and periacetabular osteotomy: retrospective matched cohort study at mean 12-year follow-up. *J Arthroplasty* 2023; 38: 323–8. 36038071 10.1016/j.arth.2022.08.034

[54] Stang A . Critical evaluation of the Newcastle-Ottawa scale for the assessment of the quality of nonrandomized studies in meta-analyses. *Eur J Epidemiol* 2010; 25: 603–5. 20652370 10.1007/s10654-010-9491-z

[55] NHLBI, NIH . Study quality assessment tools. https://www.nhlbi.nih.gov/health-topics/study-quality-assessment-tools. Accessed August 23, 2022.

[56] Novais EN, Thanacharoenpanich S, Seker A et al. Do young female dancers improve symptoms and return to dancing after periacetabular osteotomy for the treatment of symptomatic hip dysplasia?. *J Hip Preserv Surg* 2018; 5: 150–6. 29876131 10.1093/jhps/hny007 PMC5961190

[57] Jakobsen SR, Mechlenburg I, Søballe K et al. What level of pain reduction can be expected up to two years after periacetabular osteotomy? A prospective cohort study of 146 patients. *J Hip Preserv Surg* 2018; 5: 274–81. 30393555 10.1093/jhps/hny031 PMC6206701

[58] Nepple JJ, Parilla FW, Pashos GE et al. Outcomes of periacetabular osteotomy for borderline acetabular dysplasia. *J Bone Joint Surg Am* 2023; 105: 137–44. 36651889 10.2106/JBJS.22.00491

[59] Wirries N, Posselt C, Ettinger M et al. Sports activity after pelvic osteotomy for treatment of developmental dysplasia of the hip. *Orthopädie* 2022; 51: 775–80. 35394145 10.1007/s00132-022-04249-2

[60] Ricciardi BF, Fields KG, Wentzel C et al. Early functional outcomes of periacetabular osteotomy after failed hip arthroscopic surgery for symptomatic acetabular dysplasia. *Am J Sports Med* 2017; 45: 2460–7. 28617619 10.1177/0363546517710011

[61] Ricciardi BF, Mayer SW, Fields KG et al. Patient characteristics and early functional outcomes of combined arthroscopic labral refixation and periacetabular osteotomy for symptomatic acetabular dysplasia. *Am J Sports Med* 2016; 44: 2518–25. 27416990 10.1177/0363546516651829

[62] Ricciardi BF, Fields KG, Wentzel C et al. Complications and short-term patient outcomes of periacetabular osteotomy for symptomatic mild hip dysplasia. *HIP International* 2017; 27: 42–8. 27791238 10.5301/hipint.5000420

[63] Mahmoud SSS, Takla A, Meyer D et al. Arthroscopic hip surgery offers better early patient-reported outcome measures than targeted physiotherapy programs for the treatment of femoroacetabular impingement syndrome: a systematic review and meta-analysis of randomized controlled trials. *J Hip Preserv Surg* 2022; 9: 107–18. 35854801 10.1093/jhps/hnac012 PMC9291355

